# Multiparameter Profiling Distinguishes Functional Subsets of Platelets in Aging

**DOI:** 10.1093/geroni/igaf122.3627

**Published:** 2025-12-31

**Authors:** Ruth Montgomery, Adam Tanguay, Subhasis Mohanty, Ashish Shelar, Mathew Rondina, Hailong Meng, Steven H Kleinstein, Albert Shaw

**Affiliations:** Yale School of Medicine, New Haven, Connecticut, United States; Yale School of Medicine, New Haven, Connecticut, United States; Yale School of Medicine, New Haven, Connecticut, United States; Yale School of Medicine, New Haven, Connecticut, United States; Univ. Utah, Salt Lake City, Utah, United States; Yale School of Medicine, New Haven, Connecticut, United States; Yale School of Medicine, New Haven, Connecticut, United States; Yale School of Medicine, New Haven, Connecticut, United States

## Abstract

Recent studies have revealed important signaling roles in immunity for platelets, which are highly abundant in the blood and largely appreciated for their role in coagulation. Healthy older (age 65-80) and younger (age 21-35) volunteers were enrolled for studies of immune aging, and blood was collected in ACD tubes. Platelet-rich plasma was labeled fresh for profiling by mass cytometry (CyTOF) with metal-conjugated antibodies to surface markers and frozen. Randomized batches of samples were thawed, fixed, and permeabilized for labeling of intracellular markers prior to CyTOF acquisition. FCS files were bead-normalized and manually-gated platelet populations (CD45-, CD41+, CD61+) were analyzed using unsupervised FlowSOM clustering to define clusters based on surface markers. CD107b, with a role in adhesion to endothelium, characterizes distinctions between older and younger participants. The frequency of an activated cluster with low expression of CD107b was higher in platelets from older participants (10.6% vs 6.8%, p < 0.0001) and, conversely, an activated cluster with higher expression of CD107b had a lower frequency in older participants (2.9% vs 5.4%, P < 5 x10-7). Platelets from older participants also showed higher expression of CD62p, with a critical role in platelet aggregation, in both activated and resting clusters. Platelets from young participants broadly expressed higher IL-10. Notably, flow cytometry validated elevated expression of CD62p and CD63 in older adults at baseline. However, TLR-induced activation of platelets revealed blunted CD62p and CD63 responses in platelets of older individuals. Differential expression of markers and response to stimulation in platelets may contribute to aberrant immune responses during aging.

